# Associations between Macular OCT Angiography and Nonproliferative Diabetic Retinopathy in Young Patients with Type 1 Diabetes Mellitus

**DOI:** 10.1155/2020/8849116

**Published:** 2020-11-30

**Authors:** Nina C. B. B. Veiby, Aida Simeunovic, Martin Heier, Cathrine Brunborg, Naila Saddique, Morten C. Moe, Knut Dahl-Jørgensen, Hanna D. Margeirsdottir, Goran Petrovski

**Affiliations:** ^1^Center for Eye Research, Department of Ophthalmology, Oslo University Hospital, 0407 Oslo, Norway; ^2^Department of Paediatrics and Adolescent Medicine, Akershus University Hospital, 1474 Lorenskog, Norway; ^3^Department of Paediatric Medicine, Oslo University Hospital, 0407 Oslo, Norway; ^4^Institute of Clinical Medicine, Faculty of Medicine, University of Oslo, 0316 Oslo, Norway; ^5^Oslo Diabetes Research Centre, 0284 Oslo, Norway; ^6^Oslo Centre of Biostatistics and Epidemiology, Oslo University Hospital, 0372 Oslo, Norway

## Abstract

**Methods:**

OCTA of both eyes was performed in a cross-sectional study of 14 to 30-year-old individuals with at least 10-year duration of T1D and controls recruited from the Norwegian Atherosclerosis and Childhood Diabetes (ACD) study. Vessel density (VD) and foveal avascular zone (FAZ) area in the superficial and deep capillary plexus (SCP and DCP), total retinal volume (TRV), and central macular thickness (CMT) were calculated using automated software. Univariate and multivariate ordered logistic regression (OLR) models were used accordingly.

**Results:**

We included 168 control eyes and 315 T1D eyes. Lower VD in DCP (OR 0.65, 95% CI 0.51–0.83), longer diabetes duration (OR 1.51, 95% CI 1.22–1.87), and higher waist circumference (OR 1.08, 95% CI 1.02–1.14) were significantly associated with progression of NPDR. VD in SCP and DCP were significantly lower in T1D patients without diabetic retinopathy than in controls.

**Conclusions:**

Sparser VD in DCP is significantly associated with severity of NPDR, supporting that OCTA might detect the earliest signs of NPDR before it is visible by ophthalmoscopy.

## 1. Introduction

Diabetic retinopathy (DR) is the most common microvascular complication of diabetes mellitus (DM) and the leading cause of blindness in the working population of developed countries across the world. DR is asymptomatic in its early stages, and by the time visual impairment is detected, chronic and progressive pathology has already developed in the retinal microvasculature. Adolescents have a higher risk of progressing to sight-threatening retinopathy compared to adults with type 1 diabetes (T1D) and the progression may be rapid [1, 2]. Well-established risk factors for DR are poor glycemic control and longer diabetes duration. Other debated risk factors are older age, puberty, high blood pressure (BP), concomitant nephropathy, male sex, smoking, high body mass index (BMI), dyslipidemia, and celiac disease [2, 3].

Diabetic macular edema (DME) and proliferative diabetic retinopathy (PDR) are the two advanced stages of diabetic retinopathy that are the main causes of visual loss in patients with diabetes mellitus. Diabetic macular ischemia (DMI) in the absence of DME and PDR is a less commonly recognized cause of visual loss. DMI is characterized by retinal capillary loss and enlargement of the foveal avascular zone (FAZ). The understanding of the natural pathology, risk factors, and functional outcomes of DMI is limited. This has partly been because of the need for fluorescein angiography (FA) to diagnose it. With the advent of OCTA, which enables detailed depth-resolved visualization of the 3 retinal capillary plexuses (superficial, intermediate, and deep) to be evaluated independently, without the need for dye injection, the interest in studying DMI has been reignited. With widefield OCTA, this has become even more useful and will likely replace FA in the future. OCTA can measure, among others, macular vessel density and FAZ area, but it still remains unclear whether these OCTA parameters have significant functional and prognostic implications [4]. Early microvascular changes in DR such as microaneurisms, capillary dropouts such as decreased vessel density (VD), and foveal avascular zone (FAZ) enlargement are not visible by ophthalmoscopy at the early stages but can be detected by optical coherence tomography angiography (OCTA) [5]. OCTA uses the principle of “motion contrast” for the detection of blood flow and generates high-resolution cross-sectional images of the human retina in a noninvasive and reliable manner. Considering that >90% of vision loss cases can be prevented with early accurate staging and classification of DR [6, 7], OCTA plays an ever-increasing role in the diagnosis of DR and the assessment of treating options [4, 8].

To date, most studies on OCTA and DR have included adults with type 2 diabetes (T2D) or a mix of T1D and T2D [5, 9–20]. A few have focused on only T1D in adults [21, 22]. Generally, these studies have found that eyes with DR have lower VD in the SCP and/or DCP and larger FAZ than normal eyes. DR is uncommon before puberty, and there are only a few OCTA studies on children with T1D. Some of them have found no differences in macular OCTA parameters between T1D without DR and controls [23, 24], while others have found children with T1D without DR to have significantly lower VD in the DCP and larger FAZ than controls [25–27]. Puberty significantly increases the risk of DM complications; hence, adolescence is the time when efforts should be directed to screening for early signs of DR and modifiable risk factors [2]; therefore, patients aged 15-30 years with T1D with at least 10 years of diabetes duration are a very important age group. Currently, there is scarce data regarding early macular vascular changes diagnosed by OCTA in adolescents and young adults with T1D [28].

The prospective Atherosclerosis and Childhood Diabetes (ACD) study was designed to detect early atherosclerosis in young individuals with T1D by comparing them to sex- and age-matched controls. At the 10-year follow-up, the study evaluated DR by ophthalmoscopy and OCTA. In this cross-sectional part of the study, we aim to confirm whether any detectable OCTA changes exist before DR is visible for the clinician and if OCTA parameters can predict the development of NPDR in 14 to 30-year-old individuals with at least 10-year duration of T1D. We also performed this study to evaluate which of the numerous OCTA parameters have the highest diagnostic and prognostic value for OCTA to be useful in clinical practice. We also aimed to find out if any single OCTA parameters are associated with NPDR independently of traditional risk factors.

## 2. Materials and Methods

### 2.1. Study Design, Population, Eligibility Criteria, and Ethics

The individuals included in the present cross-sectional ophthalmological study performed between 2017 and 2019 were from the Norwegian Atherosclerosis and Childhood Diabetes (ACD) study, an ongoing prospective population-based study, initiated in 2006, with follow-up every 5 years. At baseline, 314 individuals with childhood-onset T1D and 120 controls aged 8-18 years were enrolled. At the 5-year follow-up, additional 15 new T1D patients and 15 new controls in the age-group 8-18 years were enrolled. The T1D patients were all on modern intensive insulin treatment with insulin pumps or basal-bolus regimens with insulin pens (≥4 daily injections), very few being also on other medication of importance, but none of significant consequence for the data. The details of the study inclusion process and examinations have been described elsewhere [29–31]. At the 10-year follow-up, all individuals enrolled in the ACD study were invited to participate in the present ophthalmological study at the Department of Ophthalmology, Oslo University Hospital; of them, 189 T1D patients and 96 controls were willing and eligible to participate [31]. Exclusion criteria were as follows: current or recent (<3 months) pregnancy, any history of ocular disease including proliferative diabetic retinopathy (PDR) and clinically significant diabetic macular edema (CDME), ocular trauma, retinal laser treatment, intravitreal injection, ocular surgery, high ametropia (spherical equivalent (SE) > ±6 D), and poor OCTA image quality. Approval for all study-specific procedures was obtained by the appropriate Regional Committee for Medical and Health Research Ethics. The described research adhered to the tenets of the Declaration of Helsinki. Written informed consent was obtained from all individuals and their parents in the case of youngsters below the age of 18.

### 2.2. Clinical and Ophthalmological Examinations

All individuals were examined according to a study protocol [29] that included diastolic (DBP) and systolic blood pressure (SBP), height, weight, waist circumference, fasting blood samples, and urine samples. Mean arterial blood pressure (MAP) was calculated as DBP+1/3 (SBP–DBP). All individuals completed a questionnaire on medical history, family history of eye disease, iris color, and medication. They underwent a routine ophthalmological examination including refraction and best-corrected visual acuity (BCVA) (Early Treatment Diabetic Retinopathy Study, ETDRS, LogMAR) at 100 LUX (Hagner Model EC1), intraocular pressure (IOP) measured with Icare tonometer (ic100, Icare, Vantaa, Finland) followed by dilation of the pupils using tropicamide 1% eye drops, only supplemented with phenylephrine 10% when needed. Mean ocular perfusion pressure (MOPP) was calculated as 2/3 (MAP–IOP) [32]. Slit-lamp examination with ophthalmoscopy, OCTA, and fundus photography of the macula and optic disc were performed after dilation. The grade of retinopathy was classified according to the International Clinical Diabetic Retinopathy (ICDR) classification system [33], and the patients with T1D were allocated into four groups: (1) T1D with no apparent NPDR (NDR), (2) mild NPDR, (3) moderate NPDR, and (4) severe NPDR. The study only comprised of individuals with nonproliferative diabetic retinopathy (NPDR) without CDME. Both eyes were included in the analyses.

### 2.3. OCTA Image Acquisition and Analysis

**Figure 1:**
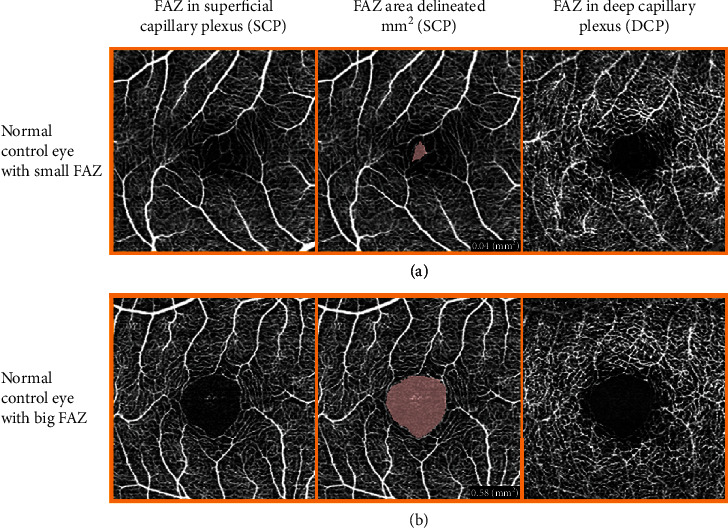
OCTA scans from two control eyes. This illustrates how the FAZ area is delineated and how different the size and shape can be in normal eyes. (a) The FAZ is 0.04 mm^2^ in the SCP, and crossing capillaries in the FAZ area makes it difficult to decide where to measure. (b) The FAZ is 0.58 mm^2^ in the SCP.

**Figure 2:**
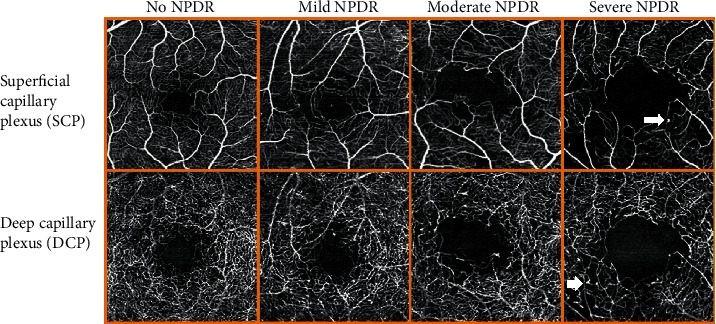
Representative 3 × 3 mm macular OCTA scans of the SCP and DCP for each ICDR level of NPDR in T1D patients. It is visible that the FAZ area increases and the vessel density decreases due to capillary dropout with the increasing level of NPDR. There are also some visible microaneurisms (arrows).

OCTA images were obtained by RS-3000 Advance AngioScan (NIDEK CO., LTD., version 1.7.0.4, Gamagori, Japan), a spectral-domain OCTA using a custom 3 × 3 mm acquisition protocol centered in the fovea. The different OCTA parameters were automatically computed by the built-in Navis-EX 1.7 software. The area of the FAZ was manually outlined in two vascular layers, SCP and DCP, and was expressed in square millimeters (mm^2^) by the software (Figure 1). The VD was analyzed in two vascular layers, the SCP and DCP, between the inner limiting membrane (ILM) and the retinal pigment epithelium (RPE) from the enface OCTA (Figure 2). The SCP consists of capillaries between the ILM and the inner plexiform layer (IPL)/inner nuclear layer (INL)+8 *μ*m. The DCP consists of capillaries in the inner nuclear layer between IPL/INL+13 *μ*m and IPL/INL+88 *μ*m. The Navis-EX software automatically computed VD, total retinal volume (TRV), and average central macular thickness (CMT) from the OCTA tomograms. The VD was expressed in mm^2^ and converted to percentage of the surface that is occupied by capillaries per area of the entire scan (9 mm^2^). We did not exclude the FAZ area when calculating the VD. TRV (mm^3^) was measured within a central 6 mm diameter circle, and CMT (*μ*m) was measured within a central 1 mm diameter circle. CDME was defined according to ETDRS as retinal thickening at or within 500 *μ*m of the macular center, hard exudates at or within 500 *μ*m of the macular center with adjacent retinal thickening, or one or more disc diameters of retinal thickening, part of which is within one disc diameter of the macular center (ETDRS study report number 1, no authors listed, [34]).

### 2.4. OCTA Quality Control

Two independent readers (NCBBV and NS) carefully evaluated each OCTA scan before the quantitative analysis. The readers were blinded to all patient characteristics. OCTA with poor image quality (SSI < 6/10) and significant image artefacts (motion lines, blurry images, and poor centration) were excluded. We also excluded those eyes that did not have all OCTA parameters measured, to avoid missing parameters.

### 2.5. Statistics and Data Analysis

Clinical characteristics are presented as means with standard deviations (SD), number (*n*) with percentages (%). Quantile-quantile (Q-Q) plots were used to check all continuous variables for normality. FAZ area in SCP and DCP was not normally distributed. We used Pearson correlation for normally distributed variables and Spearman correlation for not-normally distributed variables. We checked for multicollinearity among all the covariates with a correlation coefficient of 0.7 as a cutoff. An independent sample *t*-test was used to test for differences in mean OCTA parameters between NDR patients and controls. One-way ANOVA was used to test for differences in mean OCTA parameters between the four NPDR subgroups, and Tukey analysis was used as post hoc pairwise comparison after one-way ANOVA. In order to test which clinical and OCTA parameters were predictive of the NPDR level, we built an ordered logistic regression (OLR) model. The outcome retained four ordinal levels: no DR, mild DR, moderate DR, and severe DR. Robust standard errors were calculated while clustering on a patient level, to adjust for intraindividual correlation (since both eyes of each individual were included). For model building, we first conducted a univariable OLR analysis for each variable: gender, age, duration of diabetes, MAP, BMI, waist circumference, HbA1c, serum glucose, hemoglobin, total cholesterol, HDL cholesterol, LDL cholesterol, triglycerides, urine albumin-creatinine ratio, SE, BCVA, and IOP. All variables with a *p* < 0.05 were subsequently included in the multivariable model, to control for potential confounders. The final model was built from the multivariable model through a step-down approach. Odds ratios (OR) were reported with 95% confidence intervals (CI). FAZ in DCP and SCP, BCVA, and hemoglobin were scaled by their standard deviation to deal with convergence problems. To investigate OCTA parameters in NDR patients vs. controls, a generalized estimating equation (GEE) analysis was applied to adjust for intraindividual correlation (since both eyes of each individual were included) using the same modelling approach as described above. All statistics were performed on STATA (version 15, StataCorp LLC, TX, USA). A *p* value of <0.05 was considered statistically significant.

## 3. Results

### 3.1. Demographic and Clinical Characteristics

We examined both eyes of 285 individuals: 189 with T1D and 96 controls. After exclusion criteria were applied, 254 individuals (166 with T1D and 88 controls) and 483 eyes (315 with T1D and 168 controls) were considered suitable for analysis. Reasons for exclusion were poor OCTA image quality (*n* = 28 eyes) and poor fixation (*n* = 8 eyes), CDME (*n* = 5 eyes), PDR (*n* = 4 eyes), and spherical equivalent (SE) > 6 diopters (*n* = 2 eyes); 40 eyes could not have their OCT taken because the OCT NIDEK machine was out of order on the examination day (24 eyes with T1D without DR, 2 with moderate NPDR, and 14 control eyes).

Clinical characteristics of the patients with T1D and controls are presented in Table 1. The mean duration of T1D was 15.7 ± 3.8 years in all T1D patients. These patients had higher MAP, BMI, waist circumference, fasting blood glucose, HbA1c, and IOP and lower best-corrected visual acuity and were more myopic than the controls. Age, diabetes duration, BMI, and waist circumference increased with the increasing level of NPDR (Table 1).

### 3.2. Descriptive Analysis of OCT Parameters

Mean values of vascular and structural outcomes of OCTA are shown in Table 2. After ICDR grading, there were 239 eyes with no DR (NDR) and 58 eyes with mild, 15 eyes with moderate, and 3 eyes with severe NPDR in the T1D group. None of the controls had retinopathy. There was a large interindividual variation in the FAZ area. VD and FAZ area were higher in the DCP than in the SCP in all groups. Figure 1 shows an example of a small and a large FAZ in controls, while Figure 2 shows representative OCTA scans of SCP and DCP in NDR and mild, moderate, and severe NPDR in patients from this study.

No significant difference was found in the FAZ area in neither SCP (*p* = 0.140) nor DCP (*p* = 0.063) when comparing the NDR patients (*n* = 239 eyes) with the controls (*n* = 168 eyes). The FAZ area in both capillary plexuses showed no increase from NDR to moderate NPDR but was significantly higher in the severe NPDR group compared to the other groups (*p* < 0.001, Table 2).

**Figure 3:**
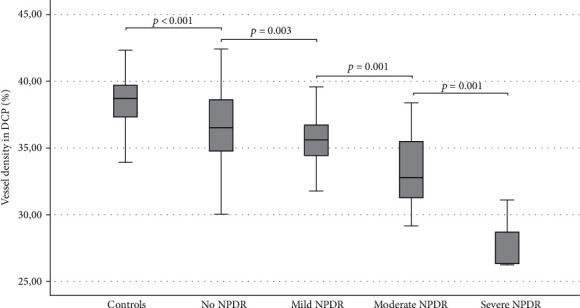
Vessel density in the deep capillary plexus is decreasing with the increasing level of NPDR. Post hoc pairwise comparison between all the subgroups after one-way ANOVA analysis shows a significant difference in vessel density between each level of NPDR.

VD in the DCP was significantly lower in the NDR patients than in controls (*p* < 0.001), and it decreased significantly with increasing grade of NPDR (*p* < 0.001, Figure 3).

VD in the SCP, TRV, and CMT were significantly lower in NDR patients than in controls, but they did not change significantly with the increasing level of NPDR (Table 2).

### 3.3. Correlations between Right and Left Eyes

OCTA parameters in right and left eyes were highly correlated: CMT (*r* = 0.92, *p* < 0.001), TRV (*r* = 0.90, *p* < 0.001), VD in SCP (*r* = 0.66, *p* < 0.001), VD in DCP (*r* = 0.77, *p* < 0.001), FAZ area in SCP (0.80, *p* < 0.001), and FAZ area in DCP (0.78, *p* < 0.001) in patients with T1D.

### 3.4. OCTA Parameters in T1D without DR vs. Controls

GEE analyses were performed to investigate clinical parameters and OCTA parameters in NDR patients vs. controls. In the final model, VD in SCP (OR 0.92, 95% CI 0.87–0.97) and DCP (OR 0.83, 95% CI 0.76–0.90), MAP (OR 1.03, 95% CI 1.01–1.06), and serum glucose (OR 1.75, 95% CI 1.53–1.99) were significantly different in NDR patients compared to controls (Table 3).

### 3.5. OCT Parameter Association with the NPDR Level

OLR analysis was performed to find out if any OCTA parameters were associated with DR independently of other traditional risk factors. With univariable analysis, VD in DCP was the only OCTA parameter that was associated with the level of NPDR (OR 0.55, 95% CI 0.44–0.71). In the multivariable model, we included the relevant variables from the univariable analyses to build the final model through a stepdown procedure. In the final model, lower VD in DCP (OR 0.65, 95% CI 0.51–0.83), longer diabetes duration (OR 1.51, 95% CI 1.22–1.87), and higher waist circumference (OR 1.08, 95% CI 1.02–1.14) were associated with the increasing level of NPDR. This means, for each 1% decrease in VD in the DCP, there was a 35% risk of jumping from one NPDR level to the next; for each year increase in diabetes duration, there was a 51% risk of jumping from one NPDR level to the next; for each 1 cm increase in waist circumference, there was an 8% risk of jumping from one NPDR level to the next, no matter what level the patient started with. Refraction was forced into the model to correct for possible magnification (Table 4).

### 3.6. Mean Ocular Perfusion Pressure (MOPP)

Of all OCTA parameters, MOPP was only correlated with VD in SCP in controls (*r* = 0.285, *p* = 0.009, Pearson correlation) and T1D patients (*r* = 0.167, *p* = 0.037). MOPP was not correlated with VD in DCP.

### 3.7. HbA1c and Waist Circumference

HbA1c was significantly correlated with waist circumference (*r* = 0.173, *p* = 0.006).

## 4. Discussion

In a population of young patients with T1D (mean age 24.3 years) imaged with macular OCTA, the VD in the DCP was found to be the only OCTA parameter associated with the increasing level of NPDR, and it could predict the development of NPDR. Lower VD in DCP, longer diabetes duration, and wider waist circumference were the three risk factors that were significantly associated with the progression of NPDR. In addition, VD in the SCP and DCP were significantly lower in T1D patients without NPDR than in controls, when adjusting for clinical confounders. VD in DCP was not associated with visual acuity. Our findings indicate that a decrease in VD in both SCP and DCP is an early process in DR and that changes in OCTA parameters are detectable before the patients have any apparent retinopathy. TRV and CMT that can also be measured with conventional OCT were not associated with the increasing level of NPDR, indicating that OCTA is superior to conventional OCT to detect changes associated with NPDR progression without macular edema. It also shows that VD in macular plexuses has a higher index to discriminate patients with T1D from individuals without T1D than FAZ area, TRV, and CMT, indicating that vascular pathology precedes thinning of the central macular area. Since the nerve fiber layer thickness was not measured in this study, it cannot be concluded whether retinal neuropathy precedes the vascular changes described. Progression of NPDR was not associated with gender, age, HbA1c, serum glucose, MAP, lipid profile, hemoglobin, and U-albumin-creatinine ratio, which may be due to the study population being young. Despite earlier studies which have shown HbA1c, after the duration of TD1, to be the most important factor in disease progression, our population showed no association between HbA1c and the level of NPDR [35, 36]. Waist circumference was strongly associated with disease progression, likely higher waist circumference reflecting better, a high level of HbA1c cumulatively over many years compared to a single blood test on the day of the eye examination. This is supported by the fact that HbA1c was significantly correlated with waist circumference in this population.

High systemic blood pressure is a well-known risk factor for retinopathy [37], which in our young population showed no association with NPDR probably because the individuals were normotensive and too young to have any significant damaging effect of it. In addition, mean ocular perfusion pressure (MOPP) was not significantly different between the NPDR groups and the controls and it was only correlated with VD in SCP; accordingly, MOPP was not an important risk factor in this population.

Our data confirm and add knowledge to previously published data by demonstrating VD in the DCP to be the most robust OCTA parameter for the differentiation of clinical stages of NPDR in young T1D patients [10, 18, 38, 39]. Other studies found lower VD in both SCP and DCP in eyes with retinopathy compared to normal eyes [9, 40]. All these earlier studies were smaller and conducted on individuals older than the ones in our study, most of them including T2D patients with comorbidities.

We used the same OLR analysis and included both eyes, almost the same clinical characteristics and OCTA parameters as a recent study [18], but their population was older (mean age 62.6 years), had a high prevalence of hypertension, included both T1D and T2D with a longer duration of diabetes (mean 14-23 years), and did not include BMI and waist circumference. They found that a higher level of HbA1c and lower VD in the DCP were associated with the increasing level of NPDR in the final model. In our younger study population, waist circumference and diabetes duration were stronger predictors for retinopathy than HbA1c. Even though the two study populations were different, both studies found that VD in DCP was the most robust OCTA parameter for detecting the level of NPDR.

Similar findings were also reported in recent publications, where the FAZ area was not different between NDR patients and normal controls, while NDR patients had lower VD limited to the DCP when compared to normal eyes. However, these studies were smaller and did not perform an OLR analysis accordingly [20, 21].

Axial length (AL) can affect the magnification of OCTA scans and may affect the quantitative results of VD. In our population, the T1D patients were more myopic than the controls, but the VD was lower in the T1D group even though myopia could have influenced the vessel density result in the other direction. In addition, refraction did not change significantly with the increasing level of NPDR. Our study found the refractive error not to be a confounder accordingly. Other studies came to the same conclusion [18, 41, 42]. Even if an algorithm was used to correct for the AL, eyes with minimal NPDR had a decreased capillary complexity and decreased vessel density compared to normal eyes, especially in the deep vascular layer in a previous study [10].

The VD was greater in the DCP than SCP in both controls and T1D patients, which is in line with other previous studies [9, 10, 14, 18]. The question remains why the DCP is more susceptible to damage than the SCP. Indeed, the same feeding retinal artery supplies the SCP in the ganglion cell layer and the DCP in the inner nuclear layer. However, anatomically, the SCP consists mainly of arterioles and venules, while the DCP of capillaries [43] makes the latter to be more susceptible to capillary closure. This theory is supported by previous histologic findings showing abnormalities to be more severe in the DCP than in the SCP [19, 44]. Also, studies have shown more microaneurisms in the DCP than in the SCP, and that the microaneurisms in the DCP contributed to the pathogenesis of macular edema [45–47]. One study has confirmed the hypothesis that diabetic macular ischemia at the level of the DCP, seen as either focally absent or low-intensity flow within the DCP on OCTA, contributes to outer retinal disruption on OCT [44]. It argues that DCP ischemia contributes to disruption of the outer retina including thinning of the outer nuclear layer and photoreceptors in eyes with DR [44]. Disturbances in vasomotion in the retinal capillary microcirculation are key factors in the development of diabetic maculopathy [48]. It has been suggested that the DCP may contribute more to the metabolic demands of photoreceptor metabolism in eyes with diabetic macular ischemia than previously thought [4, 44, 49]. Recent studies have found that ischemia or nonperfused areas in the DCP leading to lower VD as measured by OCTA is associated with abnormalities in the cone photoreceptor layer in DR as revealed by adaptive optics imaging; this suggests that the outer retinal hypoxia contributes to cone loss [49] and that complementary use of density, spacing, and packing arrangement of cones is valuable to detect early abnormalities of the parafoveal cone mosaic in adult patients with T1D. The results from this pilot study support the neurodegenerative theory, for which the retinal neuronal cells, including photoreceptors, are involved early in the course of DR [50].

Enlargement of the FAZ area is caused by the loss of capillaries in the inner vascular ring around the FAZ. We found that the FAZ area was not significantly associated with the NPDR level, but it was significantly higher in the severe NPDR group compared to other groups. A recent review paper concluded that most studies on DR found increased FAZ area in patients with diabetes compared to controls and that this was more evident in patients with advanced levels of DR [17].

According to national standards, our study was considered a big study population, and it followed a well-planned protocol in which all data were collected within a few hours in each individual. A young T1D cohort is well suited to examine retinal vascular changes due to metabolic dysregulation, since these individuals have no other vascular comorbidities such as hypertension and atherosclerosis or ocular disease, which can affect the retinal blood vessels in other ways (e.g., reduced confounders that can influence the results were avoided, so a clear influence of diabetes was obtained). Careful statistical planning was performed, and valid models were implemented to test for multiple risk factors and adjusted for potential confounders and intrapatient correlation on both eyes. The International Clinical Diabetic Retinopathy (ICDR) Disease Severity Scale was used here, since it is a more practical and valid method for use in the clinical practice than the ETDRS, thus making this study more similar to the actual clinical practice.

There are some limitations of the study as well. First, only three eyes with severe NPDR were detected, so selection bias may be possible and weak statistics due to that. Second, the current macular OCTA protocol has a small field of view; thus, we could not evaluate peripheral vascular pathology. Third, the examination time is long (between 30-45 seconds), resulting in motion artefacts, since it is hard for the patients to fixate for so long. Motion and projection artefacts may alter the interpretation of the deeper vessels, but the software has an artefact removal option that was set on default and used equally for all groups in the study. Fourth, the FAZ area was measured subjectively by the grader and could not be reliably delineated with the current NIDEK OCTA system. FAZ is irregular, difficult to measure objectively, and has considerable intergrader variability; in addition, overlap in size between the normal individuals and those with T1D was found in our study, thus not discussed further in the Results. Finally, the study was cross-sectional; therefore, it can only analyze associations between VD and DR at a given time, and not describe how VD changes over time. We excluded 36 images because of image artefacts, which may have introduced selection bias; nevertheless, we believe that it does not affect our results as the sample size is large (*n* = 483).

The traditional subjective DR grading of fundus photographs will remain clinically relevant when screening large populations, but it may fail to discover early capillary pathology which is important and only reliably detected by OCTA. We hereby suggest to make a new classification system for DR based on OCTA measurements. Automated quantification of vascular changes in the retina, primarily in the macula and on the optic disc, could translate the theoretical research usefulness of OCTA into a tool which can be easily used in “clinical practice.” OCTA may indeed be included in screening programs of patients with T1D and T2D in the future. There is evidence that vascular changes detected by the noninvasive OCTA precede the progression to more advanced levels of DR, and it may also reflect the status of the microvasculature in other organs that are only accessible by invasive biopsies. OCTA has an advantage over fluorescein angiography (FA), which only shows the superficial plexus, cannot be automatically quantified, is invasive and time consuming, and has many side effects [51–54]. Widefield OCTA will likely soon replace fluorescein angiography [55] in the near future.

In conclusion, we found that longer duration of T1D, higher waist circumference, and a sparser VD in the DCP in the macula are significantly associated with a higher odds ratio of having a worse level of NPDR. VD in DCP is associated with NPDR independently of traditional risk factors. VD in DCP measured by OCTA has a high ability to detect the earliest signs of DR, before they are actually visible by ophthalmoscopy, and it has a high ability to discriminate between different levels of NPDR. The FAZ area measured by OCTA was not a good early biomarker for DR. OCTA is a much more sensitive tool to diagnose early NPDR than conventional OCT and funduscopic ICDR grading. The objective quantification of vessel density in OCTA scans is a useful early noninvasive biomarker for the progression of DR.

## Figures and Tables

**Table 1:** Demographic and clinical characteristics of study patients (*n* = 254 individuals).

			ICDR level in patients with type 1 diabetes					
	Controls (*n* = 88)	All T1D (*n* = 166)	NDR (*n* = 113)	Mild NPDR (*n* = 40)	Moderate NPDR (*n* = 11)	Severe NPDR (*n* = 2)	*p* ^ⱡ^	*p* ^∗^
Gender male/female (male %)	41/47 (46.6%)	68/98 (41%)	48/65 (42.5%)	17/23 (42.5%)	3/8 (27.3%)	0/2 (0%)		
Age (years)	23.9 ± 3.4	24.3 ± 3.3	23.5 ± 3.4	25.3 ± 2.2	27.1 ± 1.9	27.6 ± 0.9	<0.001	0.471
Age onset of diabetes (years)		8.6 ± 3.4	8.8 ± 3.4	8.6 ± 3.1	7.3 ± 4.1	4.7 ± 2.5	0.222	
Duration of diabetes (years)		15.7 ± 3.8	14.8 ± 3.5	16.7 ± 3.3	19.8 ± 4.2	23.0 ± 1.7	<0.001	
Mean arterial blood pressure (mmHg)	85.9 ± 8.0	89.6 ± 8.0	89.1 ± 8.7	89.9 ± 5.5	93.0 ± 7.2	89.5 ± 0.23	0.473	0.001
Body mass index	23.6 ± 3.1	25.7 ± 4.5	25.2 ± 3.6	25.2 ± 4.8	31.2 ± 7.8	28.8 ± 0.02	<0.001	<0.001
Waist circumference (cm)	80.5 ± 10.0	85.2 ± 12.3	83.2 ± 9.7	86.3 ± 12.6	99.3 ± 22.7	98.7 ± 8.9	<0.001	0.002
Serum glucose (mmol/L)	4.8 ± 0.4	10.0 ± 4.3	9.9 ± 4.3	10.1 ± 4.1	9.9 ± 4.6	14.8 ± 5.8	0.462	<0.001
HbA1C (mmol/mol)	32.2 ± 2.9	64.9 ± 15.5	64.1 ± 15.3	65.3 ± 10.8	70.3 ± 27.1	78.1 ± 24.7	0.357	<0.001
Hemoglobin (g/dL)	14.4 ± 1.2	14.5 ± 1.2	14.4 ± 1.2	14.5 ± 1.3	15.2 ± 1.0	14.4 ± 0.0	0.590	0.535
Total cholesterol (mmol/L)	4.4 ± 0.7	4.5 ± 0.9	4.5 ± 0.9	4.3 ± 0.8	4.8 ± 1.0	4.6 ± 1.5	0.405	0.289
HDL cholesterol (mmol/L)	1.6 ± 0.4	1.6 ± 0.4	1.6 ± 0.4	1.5 ± 0.4	1.6 ± 0.6	1.8 ± 0.01	0.776	0.516
LDL cholesterol (mmol/L)	2.7 ± 0.7	2.8 ± 0.7	2.8 ± 0.7	2.6 ± 0.7	3.0 ± 0.7	2.6 ± 1.5	0.419	0.275
Triglycerides (mmol/L)	0.87 ± 0.4	1.01 ± 0.8	0.97 ± 0.9	1.04 ± 0.7	1.38 ± 0.9	0.85 ± 0.1	0.474	0.080
U-albumin-creatinine ratio (mg/mmol)	3.7 ± 17.0	1.0 ± 2.2	1.0 ± 2.4	1.0 ± 1.4	1.6 ± 1.6	1.4 ± 0.8	0.831	0.154
BCVA LogMAR right eye	−0.07 ± 0.07	−0.05 ± 0.07	−0.05 ± 0.07	−0.06 ± 0.06	−0.03 ± 0.11	0.00 ± 0.00	0.406	0.019
BCVA LogMAR left eye	−0.07 ± 0.08	−0.05 ± 0.07	−0.06 ± 0.07	−0.04 ± 0.07	−0.05 ± 0.09	−0.03 ± 0.06	0.420	0.014
IOP right eye (mmHg)	14.5 ± 2.7	16.1 ± 3.2	16.1 ± 3.4	16.5 ± 2.8	15.4 ± 3.8	17.7 ± 0.6	0.635	<0.001
IOP left eye (mmHg)	14.1 ± 3.0	15.9 ± 3.4	15.7 ± 3.5	16.2 ± 2.9	16.1 ± 4.3	17.7 ± 1.5	0.595	<0.001
Spherical equivalent, refraction right eye (diopters)	−0.37 ± 1.44	−1.03 ± 1.57	−0.98 ± 1.69	−1.12 ± 1.14	−1.39 ± 1.73	0.00 ± 0.71	0.651	0.001
Spherical equivalent, refraction left eye (diopters)	−0.33 ± 1.50	−0.98 ± 1.48	−0.92 ± 1.55	−1.07 ± 1.17	−1.41 ± 1.83	−0.13 ± 0.18	0.592	0.001
MOPP (mmHg)	42.8 ± 5.4	43.6 ± 5.8	43.4 ± 6.5	43.4 ± 3.9	46.7 ± 5.2	42.2 ± 0.5	0.341	0.316

Values are mean ± SD. ^∗^*p* value: independent sample *t*-test for difference between all T1D and controls. ^ⱡ^*p* value: global one-way ANOVA analysis for the difference between the NPDR subgroups.

**Table 2:** Descriptive analysis of macular OCTA parameters in controls and patients with T1D with different levels of NPDR.

		NPDR level in patients with type 1 diabetes					
OCTA parameters	Controls (*n* = 168)	NDR (*n* = 239)	Mild NPDR (*n* = 58)	Moderate NPDR (*n* = 15)	Severe NPDR (*n* = 3)	*p* ^ⱡ^	*p* ^∗^
*Vascular outcomes*							
FAZ area in SCP (mm^2^)	0.26 ± 0.09 (0.05-0.59)	0.25 ± 0.10 (0.04-0.56)	0.28 ± 0.12 (0.09-0.81)	0.29 ± 0.15 (0.08-0.70)	0.77 ± 0.58 (0.29-1.42)	*p* < 0.001	*p* = 0.14
FAZ area in DCP (mm^2^)	0.35 ± 0.09 (0.13-0.61)	0.33 ± 0.11 (0.07-0.72)	0.34 ± 0.12 (0.16-0.79)	0.39 ± 0.16 (0.18-0.73)	0.83 ± 0.55 (0.34-1.43)	*p* < 0.001	*p* = 0.063
Vessel density in SCP (%)	17.98 ± 3.52 (10.78-26.44)	16.57 ± 3.53 (9.78-28.78)	17.02 ± 2.86 (11.56-25.00)	16.94 ± 2.22 (13.67-22.33)	18.15 ± 0.34 (17.78-18.44)	*p* = 0.679	*p* < 0.001
Vessel density in DCP (%)	38.55 ± 1.83 (32.00-42.33)	36.60 ± 2.49 (30.00-42.44)	35.53 ± 1.92 (29.89-39.56)	33.23 ± 2.91 (29.22-38.44)	27.89 ± 2.79 (26.22-31.11)	*p* < 0.001	*p* < 0.001
*Structural outcomes*							
Total retinal volume, TRV (mm^3^)	9.54 ± 0.34 (8.80-10.26)	9.41 ± 0.42 (8.33-10.84)	9.52 ± 0.32 (8.25-10.14)	9.4 ± 0.40 (8.64-9.97)	9.01 ± 0.41 (8.69-9.47)	*p* = 0.082	*p* = 0.002
Central macular thickness, CMT (*μ*m)	272.74 ± 16.33 (229-309)	269.13 ± 19.80 (212-315)	269.50 ± 19.69 (232-321)	267.53 ± 28.9 (225-310)	244.67 ± 23.67 (231-272)	*p* = 0.221	*p* = 0.04

Values are mean ± SD (range). *n* = eyes. ^∗^*p* value: independent sample *t*-test for the difference between controls and T1D eyes with no NPDR (NDR). ^ⱡ^*p* value: global one-way ANOVA analysis for the difference between all the NPDR subgroups.

**Table 3:** Association between clinical risk factors and OCTA parameters in T1D patients without retinopathy vs. controls calculated by GEE analysis.

	Univariable model		Multivariable model		Final model	
	OR (95% CI)	*p*	OR (95% CI)	*p*	OR (95% CI)	*p*
*Clinical features*						
Gender	1.06 (0.83-1.35)	0.643				
Age (years)	0.99 (0.95-1.03)	0.542				
Mean arterial blood pressure (mmHg)	1.03 (1.01-1.05)	<0.001	1.03 (1.01-1.06)	0.015	1.03 (1.01-1.06)	0.007
Waist circumference (cm)	1.00 (1.01-1.03)	0.002	1.01 (0.99-1.03)	0.384		
Hemoglobin pr SD (g/dL)	1.26 (0.51-3.13)	0.617				
Serum glucose (mmol/L)	1.78 (1.57-2.03)	<0.001	1.74 (1.52-1.98)	<0.001	1.75 (1.53-1.99)	<0.001
Total cholesterol (mmol/L)	1.13 (0.97-1.13)	0.112				
HDL cholesterol (mmol/L)	0.90 (0.67-1.21)	0.498				
LDL cholesterol (mmol/L)	1.16 (0.97-1.38)	0.085				
Triglycerides (mmol/L)	1.15 (0.95-1.40)	0.157				
U-albumin-creatinine ratio (mg/mmol)	0.98 (0.95-1.01)	0.116				
Spherical equivalent (diopters)	0.84 (0.77-0.92)	<0.001	0.90 (0.78-1.04)	0.144		
Best-corrected visual acuity pr SD	1.18 (1.03-1.36)	0.020	1.09 (0.92-1.28)	0.315		
*Vascular OCTA outcomes*						
FAZ area in SCP pr SD (mm^2^)	0.88 (0.75-1.04)	0.133				
FAZ area in DCP pr SD (mm^2^)	0.86 (0.73-1.01)	0.070				
Vessel density in SCP (%)	0.93 (0.90-0.97)	<0.001	0.93 (0.88-0.99)	0.014	0.92 (0.87-0.97)	0.002
Vessel density in DCP (%)	0.78 (0.73-0.83)	<0.001	0.84 (0.76-0.92)	<0.001	0.83 (0.7-0.90)	<0.001
*Structural OCT outcomes*						
Total retinal volume (mm^3^)	0.59 (0.43-0.82)	0.002	0.73 (0.40-1.32)	0.294		
Central macular thickness (*μ*m)	0.99 (0.98-1.00)	0.044	1.00 (0.99-1.01)	0.931		

**Table 4:** The associations of clinical risk factors and OCTA parameters with the increasing level of NPDR calculated as odds ratios with ordered logistic regression analysis.

	Univariable model		Multivariable model		Final model	
	OR (95% CI)	*p*	OR (95% CI)	*p*	OR (95% CI)	*p*
*Clinical features*						
Gender	2.51 (0.62-10.2)	0.198				
Age (years)	1.90 (1.39-2.60)	<0.001	1.31 (0.99-1.74)	0.061		
Duration of diabetes (years)	1.74 (1.36-2.22)	<0.001	1.38 (1.11-1.73)	0.004	1.51 (1.22-1.87)	<0.001
Mean arterial blood pressure (mmHg)	1.06 (0.97-1.15)	0.182				
Body mass index	1.24 (1.05-1.48)	0.014				
Waist circumference (cm)	1.12 (1.05-1.20)	0.001	1.07 (1.01-1.13)	0.014	1.08 (1.02-1.14)	0.005
HbA1C (mmol/mol)	1.57 (0.96-2.56)	0.070				
Serum glucose (mmol/L)	1.06 (0.90-1.25)	0.456				
Hemoglobin pr SD (g/dL)	1.26 (0.51-3.13)	0.617				
Total cholesterol (mmol/L)	0.98 (0.46-2.09)	0.967				
HDL cholesterol (mmol/L)	0.92 (0.18-4.82)	0.920				
LDL cholesterol (mmol/L)	0.81 (0.32-2.06)	0.659				
Triglycerides (mmol/L)	1.74 (0.78-3.89)	0.180				
U-albumin-Creatinine ratio (mg/mmol)	1.13 (0.84-1.51)	0.295				
Spherical equivalent (diopters)	0.90 (2.29-1.37)	0.619	0.98 (0.65-1.48)	0.913	0.86 (0.58-1.29)	0.477
Best-corrected visual acuity pr SD	2.29 (1.11-4.71)	0.025	1.59 (0.83-3.02)	0.159		
IOP (mmHg)	1.08 (0.89-1.30)	0.443				
*Vascular OCTA outcomes*						
FAZ area in SCP pr SD (mm^2^)	1.83 (0.96-3.50)	0.068				
FAZ area in DCP pr SD (mm^2^)	1.67 (0.89-3.12)	0.108				
Vessel density in SCP (%)	1.10 (0.93-1.30)	0.258				
Vessel density in DCP (%)	0.55 (0.44-0.71)	<0.001	0.67 (0.52-0.85)	0.001	0.65 (0.51-0.83)	<0.001
*Structural OCT outcomes*						
Total retinal volume, TRV (mm^3^)	0.92 (0.18-4.77)	0.925				
Central macular thickness, CMT (*μ*m)	0.98 (0.95-1.02)	0.367				

## Data Availability

All data will be made available and deposited according to journal policy.
